# Selective masking and demasking for the stepwise complexometric determination of aluminium, lead and zinc from the same solution

**DOI:** 10.1186/1752-153X-2-4

**Published:** 2008-03-06

**Authors:** Nijhuma Kayal, Nahar Singh

**Affiliations:** 1 Analytical Chemistry Section, Central Glass and Ceramic Research Institute, 196, Raja S.C. Mullick Road, Kolkata-32, India; 2 Analytical Chemistry section, National Physical laboratory, Dr K.S. Krishnan Marg, New Delhi-12, India

## Abstract

**Background:**

A complexometric method based on selective masking and de-masking has been developed for the rapid determination of aluminium, lead and zinc from the same solution in glass and glass frit samples. The determination is carried out using potassium cyanide to mask zinc, and excess disodium salt of EDTA to mask lead and aluminium. The excess EDTA was titrated with standard Mn(II)SO_4 _solution using Erichrome Black-T as the indicator. Subsequently selective de-masking agents – triethanolamine, 2,3-dimercaptopropanol and a formaldehyde/acetone mixture – were used to determine quantities of aluminium, lead and zinc in a stepwise and selective manner.

**Results:**

The accuracy of the method was established by analysing glass certified reference material NBS 1412. The standard deviation of the measurements, calculated by analysing five replicates of each sample, was found to be less than 1.5% for the method proposed.

**Conclusion:**

The novelty of the method lies in its simplicity and accuracy afforded by there not being a need for a prior separation or instrumentation. The proposed method was found to be highly selective for the precise determination of aluminum, zinc and lead in the routine analysis of glass batch and allied materials.

## 1. Background

The production of glasses and glass frits has considerably increased in recent decades owing to expansion of such materials' applications in modern science and technology. The composition of glass and glass frits dictates their applications, with physico-chemical properties like optical, thermal expansion, electrical, flow ability and chemical resistance varying [1]. In general the major to minor constituents in glass frit and glass are respectively: SiO_2_, Na_2_O, Al_2_O_3_, PbO, TiO_2_, ZnO, MgO, BaO, B_2_O_3_, F, CaO, and ZrO_2 _; while the trace elements like Cd, Co, Ni, Fe, Mn, Sn, Cr are sometimes found to be present, when employed in certain applications [2]. Lead, aluminium and zinc are often present together in optical glasses and low melting glass frits [3]. Lead bearing glasses possess low softening points and are used to join one glass to another or a metal to a glass frit [4]. The function of aluminium in frits is to reduce atmospheric moisture attack in an acidic environment and increase the viscosity and softening point of the frit. Zinc enhances the optical, thermal and electrical properties of glass and glass frit [4,5].

Complexometry is a very useful method for the determination of major quantities of metals present in various combinations. The literature shows that analyses of such materials are based on separation, which is laborious and time consuming [6-9]. The conventional methods for the determination of aluminium, zinc and lead are based on complexometry using EDTA [6,7], colorimetry [8] or polarographic approaches [9]. In all these procedures the quantity of the element is determined individually using separate aliquots with the interfering elements in some cases being separated. Dasgupta *et al *[10,11] described another method, based on gravimetric, complexometric and instrumental techniques, which proved to be complex and lengthy. More recently several workers have reported different methods [12-16] for the determination of these elements in glass and allied materials by inductively coupled plasma atomic emission spectroscopy (ICP-AES) and atomic absorption spectroscopy (AAS) [13-16]. Undoubtedly ICP-AES and AAS are the preferred methods although the relevant instrumentation is costly. When using such instruments the elements either need to be separated, extracted or masked from other interfering elements. Several instrumental parameters must be controlled. Therefore rapid and precise determination of aluminum, zinc and lead in the same solution by complexometric method is a challenge as a regards quality control.

In the complexometric titrations, masking and demasking agents have been used from past decades. Pribil and his coworkers demonstrated wide applications of using more than one masking agent and combined masking and demasking agents in complexometric methods [17,18] for large quantities of cations when present together. Potassium cyanide is an effective masking agent for large quantities of cations; similarly triethanolamine and 2,3 dimercaptopropanol have been employed in several studies [17-20].

In light of these previous studies, we have attempted to determine the quantities of aluminium, zinc and lead in the same solution in a selective and quantitative way by masking zinc with potassium cyanide, aluminium and lead with EDTA, and then de-masking the respective complexes using a formaldehyde:acetone mixture, triethanolamine and 2,3-dimercaptopropanol. The process is simple, accurate and does not require separation and extraction or costly instrumentation.

## 2. Results and discussion

The conventional complexometric method [10,11] for the determination of aluminium in silicates involves back titration of EDTA with zinc acetate at pH 5.3, under which conditions all other R_2_O_3 _group elements (titanium, zirconium) are complexed by EDTA. Therefore an appropriate correction is needed to obtain the exact quantity of aluminium. In this study the complexation of the metal with EDTA is carried out at higher pH (10) and at lower temperature (10°C). Under these conditions trace to small quantities (0.05 to 5 mg) of titanium and zirconium do not interfere. For the selective demasking of aluminium, we employed triethanolamine, which also reacts with iron. To avoid interference by iron, ascorbic acid was added before the addition of KCN, ensuring that iron and manganese (if present) are reduced and complex with cyanide. However, for large quantities of chromium and nickel, the colouration of the cyanide complexes obscure the end point in the subsequent titration with manganese sulfate. Normally glass contains trace amounts of chromium, nickel and cobalt, which do not interfere because of the formation of cyanide complex. In general glass frit samples contain small quantities of magnesium, which form Mg-EDTA complexes and may react with manganese releasing the magnesium ion, causing difficulty in the end point detection. However this type of replacement reaction occurs at higher temperatures, thus EDTA complexes of Ca and Mg remains in solution without interfering and for this reason it is necessary to carry out the reaction at low temperatures. For the quantitative demasking of lead, 2,3-dimercaptopropanol was found to be suitable. It acts by eliminating lead by converting non-interfering complexes, thereby allowing liberated EDTA to be measured. Yet when the percentage of lead is high, a yellow colouration may appear which hinders the end point detection. This problem can be overcome by the addition of a small amount of potassium hydroxide solution. This masking and demasking technique can also avoid the interference of barium during the titration of lead. For selective demasking of zinc from its cyanide complex it has been observed that addition of a formaldehyde:acetone mixture is more effective than that of formaldehyde alone. The result obtained by following the procedure is in good agreement with the known quantities of aluminium, lead and zinc taken in a synthetic solution (Table 1) indicating good accuracy. This method is successfully applied to the determination of aluminium, lead and zinc in glass, and glass frit samples and one standard reference material (SRM). The elements are also determined using the existing method, *i.e*. aluminium by complexometry (back titration with zinc acetate) and zinc and cadmium by polarography [10]. The average of five determinations with standard deviations is presented in Table 2 and 3 for reference material (NBS 1412) and glass samples respectively. The analytical results in Tables 2 and 3 reveal that there is good agreement between the proposed and existing methods.

**Table 1:** Determination of aluminium, lead and zinc in synthetic solutions.

Sample	Amount taken/mg			Found by the proposed method/mg			Recovery/%		
	
	Al	Pb	Zn	Al	Pb	Zn	Al	Pb	Zn
1.	20	50	20	19.88 ± 0.12	49.95 ± 0.17	20.02 ± 0.07	99.4	99.9	100.1
2.	10	40	15	10.02 ± 0.07	40.16 ± 0.20	14.97 ± 0.11	100.2	100.4	99.8
3.	5	30	10	4.97 ± 0.02	30.15 ± 0.19	10.06 ± 0.05	99.4	100.5	100.6
4.	5	5	5	4.98 ± 0.03	5.02 ± 0.5	5.03 ± 0.07	99.6	100.4	100.6

**Table 2:** Determination of aluminium, lead and zinc in multicomponent glass reference material NBS 1412.

Sample	Constituents	Certified values of NBS 1412	Results obtained by proposed method	Result obtained by the standard method^10^
1.	Al_2_O_3_%	7.52	7.54 ± 0.11	7.59 ± 0.13
2.	PbO %	4.40	4.42 ± 0.08	4.38 ± 0.09
3.	ZnO %	4.48	4.47 ± 0.08	4.42 ± 0.09

**Table 3:** Determination of aluminium, lead and zinc in glass samples using proposed and standard methods.

Constituents	Value (Mean % of five determinations) using the proposed method			Value (%) found using the standard method ^10^		
	Sample no. 1	Sample no. 2	Sample no. 3	Sample no. 1	Sample no. 2	Sample no. 3
Al_2_O_3_	12.57 ± 0.02	1.16 ± 0.09	3.63 ± 0.09	12.59 ± 0.09	1.14 ± 0.09	3.62 ± 0.09
PbO	42.60 ± 0.27	40.36 ± 0.06	42.22 ± 0.35	42.68 ± 0.09	40.38 ± 0.09	42.25 ± 0.09
ZnO	11.14 ± 0.19	9.84 ± 0.02	19.53 ± 0.11	11.12 ± 0.09	9.85 ± 0.09	19.56 ± 0.09

## 3. Conclusion

Optical, electrical, mechanical and other properties of glass and other allied products are largely dependent upon chemical composition. Therefore a suitable analytical method is essential for the precise determination of the concentration of constituents in desired products. The proposed method is highly selective for the determination of aluminium, zinc and lead for routine analysis of glass batch and allied materials with good precision. In addition, the analyses have been carried out from a single aliquot, and analytical results for the standard glass sample compare favourably with certified values.

## 4. Experimental

### 4.1 Apparatus

Calibrated pipettes and volumetric flasks supplied by Borosil Glass Works Ltd India were used. The digestion process was carried out on a Laminar flow bench equipped with an appropriate ventilation system.

### 4.2 Reagents

Hydrofluoric acid 40% (m/m), nitric acid (16 N), ammonia solution (NH_4_OH), triethanolamine 30% (v/v), potassium cyanide solution 20% (m/v), 2,3-dimercaptopropanol, formaldehyde solution, acetic acid, ascorbic acid 98% of AR/GR grade and all other standard chemicals supplied by E. Merck (Germany) were used. De-ionized water (18 mega ohm resistivity) prepared from the Millipore milli-Q water purification system, USA, was used throughout.

### 4.3 Standard solutions

#### 4.3.1 Standard zinc solution, 0.01 M

0.656 g of zinc metal (99.99% purity) was dissolved in hydrochloric acid and diluted to 1 L with distilled water.

#### 4.3.2 Standard EDTA solution, 0.01 M

3.744 g of the disodium salt of EDTA were dissolved in deionized (DI) water and diluted to 1 L. The stock solution was standardised according to the conventional method [6] with standard 0.01 M zinc solution using EBT as the indicator.

#### 4.3.3 Mn(II)SO_4 _solution 0.01 M and 0.005 M

For the preparation 0.01 M and 0.005 M Mn(II)SO_4 _solutions, respectively, 1.7 g and 0.85 g of 1-hydrate were dissolved in water and diluted to 1 L with water. The stock solution of 0.01 M Mn(II)SO_4 _was standardised against standard aluminium according to the conventional method using EBT indicator. Similarly 0.005 M Mn(II)SO_4 _solution was standardised against standard lead solution according to the conventional method [6] using EBT indicator.

#### 4.3.4 Lead nitrate Solution, 0.01 M

For the 0.01 M stock solution, 3.312 g of lead nitrate were dissolved in water and acidified with a few drops of HNO_3_, before being diluted to 1 L with DI water.

#### 4.3.5 Standard aluminium solution, 0.025 M

0.6745 g of polished aluminum foil were cleaned with absolute alcohol and then dissolved in 25 mL of hydrochloric acid and 150 mL of DI water, before being further diluted to 500 mL.

#### 4.3.6 Erichrome Black-T

This was prepared by dissolving 0.12 g of EBT and 1.2 g hydroxylamine hydrochloride in ethanol.

### 4.4 Preparation of the sample solution

0.5 g of a well ground sample obtained after loss on ignition (100 ± 5°C) was put into a cleaned teflon basin, moistened with a few drops of water, before the addition of 2 mL of HNO_3 _and 10 mL of 40% HF acid. The Teflon basin containing whole components was evaporated to dryness on a hot plate and the process repeated several times to ensure the total evaporation of silica as SiF_4_. The residual mass was then dried several times with 10 mL of DI water to remove the acids. Finally the residue was dissolved with 5 mL of HNO_3 _and diluted to 250 mL with DI water. Subsequently, 25 mL of stock solution were taken in a conical flask and diluted to 100 mL to which was added a known excess amount of 0.01 M EDTA solution and 0.1 g of ascorbic acid, before being boiled on a hot plate for 1 minute. To this solution 25 mL of 20% potassium cyanide solution were added and immediately 10 mL of concentrated ammonium hydroxide were added to prevent the formation of HCN. During work with KCN gloves and masks must be used as a precaution. The total solution was cooled to 10°C in an ice bath, and excess EDTA was titrated against standard 0.01 M manganese solution, with a few drops of a 0.1% alcoholic solution of Erichrome Black T as indicator. At the end point the blue colouration was seen to change to red. The total volume of the EDTA consumed in the reaction corresponded to the sum of all the constituents which complexed with EDTA.

## 5. Procedure

### 5.1 Determination of Al

To determine aluminium, 20 mL of triethanolamine and 5 mL of hydroxyl amine 10% (w/v) were added to the solution remaining after the above titration, before being boiled for 1 minute. The liberated EDTA was then titrated with standard 0.01 Mn(II)SO_4 _solution using Erichrome Black-T as the indicator. At the end point a blue colouration was seen to change to red. The consumed manganese solution was equivalent to the aluminium content of the sample.

### 5.2 Determination of lead

After the titration of aluminium, 5 mL of 20% alcoholic solution of 2,3-dimercaptopropanol were added with slow swirling. The red colouration was seen to change to blue. Again liberated EDTA was titrated with 0.005 M manganese using Erichrome Black-T. At the end point the blue colouration was seen to change to a sharp red wine colour. The consumed manganese solution corresponded to the lead content of the sample.

### 5.3 Determination of zinc

After the titration of lead, zinc was quantitatively de-masked from its cyanide complex by the addition of (3:1) 20 g of 4% formaldehyde:acetone solution. Then the liberated zinc was titrated with a 0.01 M EDTA solution using EBT as the indicator. The end point was indicated by a sharp colour change from red to blue. After titration addition of a formaldehyde:acetone mixture was repeated followed by a second titration, until the solution no longer turned red. The total amount of EDTA consumed corresponded to the zinc content of the sample.
